# ﻿A new species of *Trisiniotus* Jeannel from Mao-shan, East China (Coleoptera, Staphylinidae, Pselaphinae)

**DOI:** 10.3897/zookeys.1095.81076

**Published:** 2022-04-13

**Authors:** Ting Feng, Zi-Wei Yin

**Affiliations:** 1 Shanghai Zoological Park, 2381 Hongqiao Road, Changning District, Shanghai 200335, China Shanghai Zoological Park Shanghai China; 2 Laboratory of Systematic Entomology, College of Life Sciences, Shanghai Normal University, 100 Guilin Road, Xuhui District, Shanghai 200234, China Shanghai Normal University Shanghai China

**Keywords:** China, Jiangsu, new species, taxonomy, *Trisiniotus*

## Abstract

The genus *Trisiniotus* Jeannel of the pselaphine tribe Batrisini comprises two species distributed in North India and southern Myanmar. Here, a third species, *T.taoismus* Feng & Yin **sp. nov.**, is described from Mao-shan, Jiangsu Province, East China. The new species can be readily distinguished from both congeners by the unmodified male antennae.

## ﻿Introduction

In his review of the pselaphine fauna of North India, Jeannel (1960) established a number of batrisine genera that, from a modern point of view, lack adequate justification. The genus *Trisiniotus* Jeannel certainly falls into this category. No specific comments were provided on generic characteristics, nor was the genus compared to potentially related genera. In the key to genera (Jeannel 1960: 409), *Trisiniotus* was coupled with *Batristhenes* Jeannel by the shared absence of a median longitudinal sulcus on the pronotum, and separated from the latter by the modified antennae and different shapes of the head and aedeagus. The absence (or loss) of a median sulcus on the pronotum appears to be homoplastic in a number of Asian batrisine genera, as pointed out by Nomura (1991: 11), and the presence of antennal modifications is a common state in numerous batrisine groups. Both characters can hardly be considered as bearing any systematic significance above the species level. The constricted basal capsule of the aedeagus of *Trisiniotus* may indicate, to a certain degree, close relationships with the Japanese species of *Batriscenaulax* Jeannel and *Physomerinus* Jeannel, which have the same type of the aedeagus and are separated from each other mainly by different locations of the male sexual characters (Jeannel 1958). The type species of *Trisiniotus*, *T.nodicornis* Jeannel, was reported from Dehradun in Uttarakhand, India and remained the sole member of the genus until Nomura and Aung (2020, 2021) recently identified *Batrisusnitidulus* Motschulsky (type locality: “Ind. or.”) among material from southern Myanmar, and transferred the species to *Trisiniotus*. A third, distinct but unnamed species that has an enlarged antennomere 9 and a medially sulcate pronotum was reported by Nomura et al. (2013) from Kaeng Krachan National Park, southern Thailand. Putting aside the uncertain generic placement of *Trisiniotus*, both named species are easily recognizable by the swollen antennomere 10 in the male.

In August 2020, we organized a collecting trip to Mao-shan Scenic Area, Jiangsu Province in the hope of finding additional material of a distinctive clavigerite beetle (described as *Archiclavigergaofani* Yin, Hlaváč & Cuccodoro in Yin et al. 2020) collected in the city of Changzhou and sent to the junior author shortly before the trip. We failed to find this beetle, probably owing to it being the wrong season. Nevertheless, a small series of pselaphines were collected by sifting the leaf litter layer in forests along the Mao-shan Mountain range. Among this material we recognized a distinct species of Batrisini, which is evidently related to the two *Trisiniotus* species from India and Myanmar based on the shape of the pronotum and aedeagus. As no attempt is made here to clarify the relationships of *Trisiniotus* with potentially related groups, we simply describe the new species and place it as a member of *Trisiniotus*.

## ﻿Material and methods

The type material of the new species described in this paper is deposited in the Insect Collection of Shanghai Normal University, Shanghai (SNUC). The label data of the material are quoted verbatim.

Dissected parts were mounted in Euparal on plastic slides pinned with the specimen. The habitus image of the beetle was taken using a Canon 5D Mark III camera with Canon MP-E 65 mm f/2.8 1–5 × Macro Lens, with a Canon MT-24EX Macro Twin Lite Flash as the light source. Images of the morphological details were produced using a Canon G9 camera mounted to an Olympus CX31 microscope under reflected or transmitted light. Zerene Stacker (version 1.04) was used for image stacking. All images were modified and grouped into plates using Adobe Photoshop CC 2020.

Measurements were taken as follows: total body length was measured from the anterior margin of the clypeus to the apex of the abdomen; head length was measured from the anterior margin of the clypeus to the head base, excluding the occipital constriction; head width was measured across the eyes; the length of the pronotum was measured along the midline; the width of the pronotum is its maximum width; the length of the elytra was measured along the suture; the width of the elytra was measured as the maximum width across both elytra; the length of the abdomen is the length of the dorsally exposed part of the abdomen along its midline; the width of the abdomen is its maximum width. Abdominal tergites and sternites are numbered following Chandler (2001), in Arabic (starting from the first visible segment) and Roman (reflecting true morphological position) numerals, e.g., tergite 1 (IV), or sternite 1 (III). Paired structures in the description of the new species are treated as singular.

### ﻿Taxonomy

#### ﻿ Trisiniotus taoismus

Taxon classificationAnimaliaColeopteraStaphylinidae

Feng & Yin sp. nov.

D8E7A2BB-8914-5299-BF9E-B1CFDF2440D8

http://zoobank.org/C2995259-9A1F-42FF-B22D-A0169BB25162

**Figure 1.**
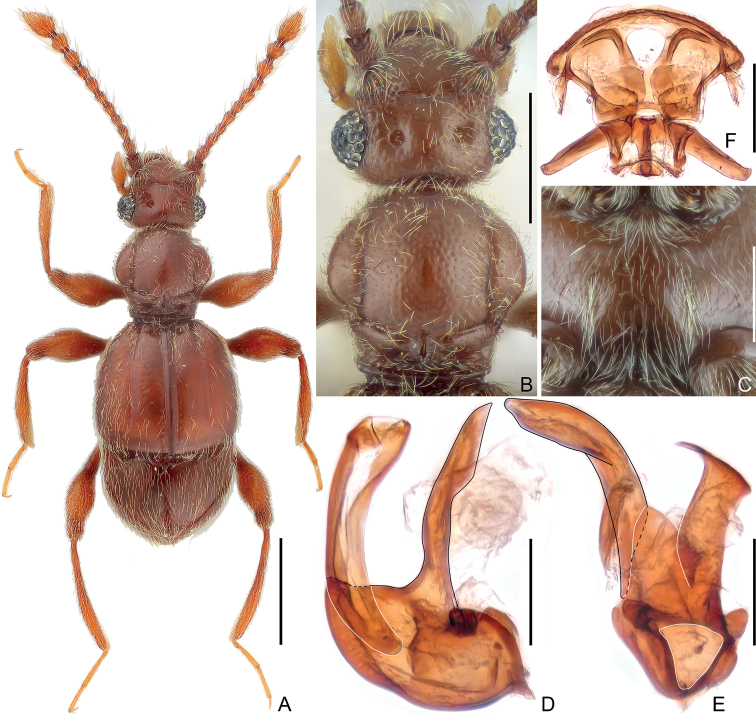
Morphological characters of *Trisiniotustaoismus* sp. nov. **A** dorsal habitus **B** head dorsum and pronotum **C** central part of metaventrite **D, E** aedeagus, lateral (**D**) and ventral (**E**) **F** female genitalia. Scale bars: 0.5 mm (**A**); 0.3 mm (**B**); 0.2 mm (**D–F**).

**Figure 2.**
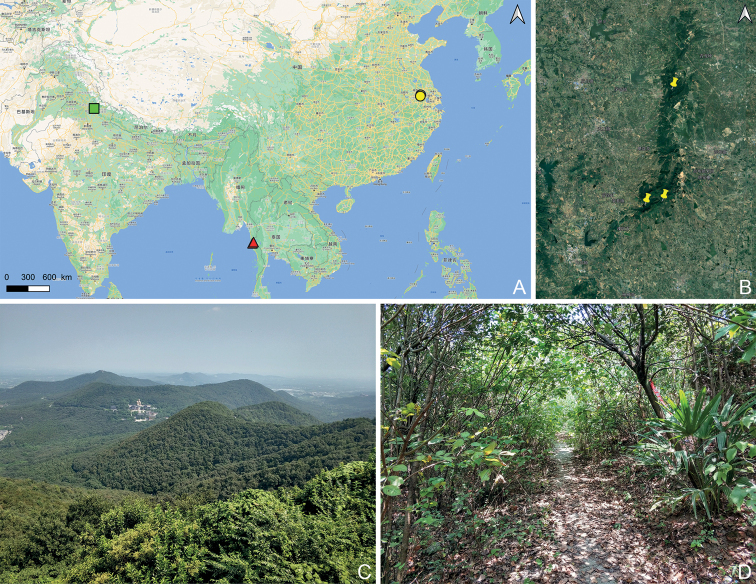
Distribution (**A, B**) and habitat (**C, D**) of *Trisiniotus*. **A** distribution of *T.taoismus* sp. nov. (circle), *T.nitidulus* (triangle), and *T.nodicornis* (square) **B** distribution of *T.taoismus* sp. nov. in the Mao-shan mountains **C** environment of Mao-shan **D** habitat of *T.taoismus* sp. nov.

Chinese common name: 道隐沟蚁甲 Figs 1 , 2

##### Type material

**(15 exx.). *Holotype***: China: ♂, ‘China: Jiangsu, Jurong City, Mao Shan, 31°47'41.99"N, 119°18'43.38"E, leaf litter, sifted, 140 m, 24.viii.2020, Ting Feng leg., 江苏句容市茅山风景区’ (SNUC). ***Paratypes***: China: 6 ♂♂, 3 ♀♀, same data as that of holotype; 1 ♂♂, 2 ♀♀, ‘China: Jiangsu, Jurong City, Yaji-shan, 31°39'24.06"N, 119°17'253.71"E, leaf litter, sifted, 100 m, 23.viii.2020, Zi-Wei Yin leg., 江苏句容市丫髻山脚; 2 ♀♀, China: Jiangsu, Jurong City, nr. Wawu-shan, 31°39'6.28"N, 119°16'20.99"E, leaf litter, sifted, 100 m, 22.viii.2020, Ting Feng leg., 江苏句容市瓦屋山上杆湖农庄 (all SNUC).

##### Diagnosis.

**Male.** Body length approximately 1.9 mm. Head sub-rectangular at base; vertex with large and setose foveae, with transverse sulcus at anterior portion; antenna elongate; antennomeres more or less elongate, lacking modifications. Pronotum lacking a median longitudinal sulcus. Discal stria of elytron long, extending posteriorly to approximately apical 3/4 of elytral length. Mesotibia with small apical spine. Metaventrite with setose admesal longitudinal ridges. Tergite 1 (IV) predominantly large, dorsally longer than 2–4 (V–VII) combined, lacking modifications. Aedeagus strongly asymmetrical; median lobe with restricted basal capsule and triangular foramen, ventral stalk erect, narrowing towards apex in lateral view; dorsal lobe narrowed at base, broadened towards apex. **Female.** Body length approximately 1.8 mm, legs and metaventrite lacking modifications, genitalia as in Fig. 1F.

##### Description.

**Male.** Body (Fig. 1A) length 1.88–1.92 mm; color reddish-brown, tarsi and mouthparts lighter. Dorsal surface of body covered with short pubescence.

Head (Fig. 1B) roundly triangular, sub-rectangular at base, much wider than long, length 0.37–0.39 mm, width across eyes 0.44 mm; vertex finely punctate, with large, setose vertexal foveae (dorsal tentorial pits), with transverse sulcus at apical portion of vertex, mediobasal carina thin and faint; antennal tubercles weakly raised; frons slightly impressed medially, confluent with clypeus; clypeus smooth, its anterior margin carinate and moderately raised; ocular-mandibular carina complete, distinct, carina branched below eye, extended ventrally and then anteriorly to posteroventral articulation of mandible. Venter with single, small gular fovea (posterior tentorial pit), with distinct median carina extending from fovea anteriorly to mouthparts. Eyes greatly prominent, each composed of approximately 35 large ommatidia. Antenna moderately elongate, length 0.94–0.97 mm, simple, club loosely formed by moderately enlarged apical three antennomeres; antennomere 1 thick, subcylindrical, 2–7 each elongate, 8 shortest, 9 much longer and broader than 8, 10 as long as and slightly wider than 9, 11 longest, shorter than 9 and 10 combined, sub-conical.

Pronotum (Fig. 1B) approximately as long as broad, length 0.45–0.46 mm, width 0.46–0.47 mm, widest at middle; sides rounded; disc slightly convex, finely punctate, median longitudinal sulcus absent, semi-circular lateral sulci extending from dorsal surface laterally and posteriorly and then fused with lateral ends of antebasal sulcus; lacking median antebasal fovea, with short mediobasal impression, antebasal tubercles small, lateral antebasal foveae connected by transverse antebasal sulcus; outer and inner pairs of basolateral foveae distinct. Prosternum with anterior part as long as coxal part, with small lateral procoxal foveae; hypomeral ridge short, present only at base, with a lateral antebasal hypomeral impression; margin of coxal cavity weakly carinate.

Elytra much wider than long, length 0.62–0.63 mm, width 0.69–0.70 mm; each elytron with two large, asetose basal foveae, lacking a subbasal fovea; discal stria long, carinate, extending from outer basal fovea to approximately apical 3/4 of elytral length; humerus rounded, weakly prominent, subhumeral fovea absent, with sulcate marginal stria from below middle to posterior margin of elytron. Metathoracic wings fully developed.

Mesoventrite short, demarcated from metaventrite by ridged anterior edges of impressed areas where lateral mesocoxal foveae situated at mesal ends of impressions, with pair of thin admesal carinae; setose median mesoventral foveae widely separated, lateral mesoventral foveae large and setose, broadly forked internally; intercoxal process short. Metaventrite (Fig. 1C) broadly impressed at middle, with setose admesal longitudinal ridges; with large, setose lateral mesocoxal foveae and a pair of smaller, setose lateral metaventral foveae, posterior margin broadly emarginate, with narrow split at middle.

Legs moderately elongate; mesotibia with small spine at apex.

Abdomen compressed, widest at lateral margins of tergite 1 (IV), length 0.47–0.50 mm, width 0.64–0.68 mm. Tergite 1 (IV) in dorsal view longer than 2–4 (V–VII) combined, lacking basal sulcus, with one pair of basolateral foveae and a short discal carina; tergites 2–4 each with one pair of small basolateral foveae, 4 as long as 2 and 3 combined along middle, 5 (VIII) semicircular, posterior margin roundly emarginate at middle. Sternite 2 (IV) with mediobasal and two basolateral foveae, with a pair of short lateral carinae; midlength of sternite 2 as long as 3–5 (V–VII) combined, 3–5 each short at middle, lacking fovea, 6 (VIII) transverse, posterior margin emarginate at middle, 7 (IX) membranous or absent.

Aedeagus (Fig. 1D, E) 0.30 mm long, strongly asymmetrical; median lobe with constricted basal capsule and small, roundly triangular foramen, ventral stalk erect, in lateral view broadest anterior to middle and then narrowing towards apex; dorsal lobe approximately as long as ventral stalk, narrowed at base and broadening towards apex; parameres reduced to single broad membranous structure.

**Female.** Similar to male in external morphology; antenna shorter; each compound eye composed of approximately 30 ommatidia; elytra constricted at bases, humerus not prominent; metathoracic wings absent; mesotibia lacking apical spine, metaventrite lacking admesal ridges. Measurements (as for male): body length 1.79–1.83 mm; length/width of head 0.37/0.42–0.43 mm, pronotum 0.41–0.43/0.45 mm, elytra 0.56–0.57/0.69 mm; abdomen 0.52–0.53/0.65 mm; length of antenna 0.85–0.87 mm; maximum width of genitalia (Fig. 1F) 0.23 mm.

##### Comparative notes.

*Trisiniotustaoismus* sp. nov. shares with its two congeners the lack of a median longitudinal sulcus on the pronotum, as well as a similar aedeagus. This species can be readily separated by the simple male antennomere 10, which is swollen in both species from India and Myanmar.

##### Distribution.

East China: Jiangsu (Fig. 2A, B).

##### Bionomics.

All individuals were collected by sifting the leaf litter layer in broad-leaved forests (Fig. 2C, D).

##### Etymology.

The specific epithet reflects that Mao-shan is a Taoist sacred mountain in eastern China.

## Supplementary Material

XML Treatment for Trisiniotus taoismus
